# Microfluidic Single-Cell Study on *Arabidopsis thaliana* Protoplast Fusion—New Insights on Timescales and Reversibilities

**DOI:** 10.3390/plants13020295

**Published:** 2024-01-18

**Authors:** Thorsten Seidel, Philipp Johannes Artmann, Ioannis Gkekas, Franziska Illies, Anna-Lena Baack, Martina Viefhues

**Affiliations:** 1 Dynamic Cell Imaging, Faculty of Biology, Bielefeld University, 33615 Bielefeld, Germany; 2 Experimental Biophysics and Applied Nanosciences, Faculty of Physics, Bielefeld University, 33615 Bielefeld, Germany

**Keywords:** protoplast, *Arabidopsis thaliana*, induced cell fusion, microfluidics, poly(ethylene glycol), single-cell analysis

## Abstract

Plant cells are omnipotent and breeding of new varieties can be achieved by protoplast fusion. Such fusions can be achieved by treatment with poly(ethylene glycol) or by applying an electric field. Microfluidic devices allow for controlled conditions and targeted manipulation of small batches of cells down to single-cell analysis. To provide controlled conditions for protoplast fusions and achieve high reproducibility, we developed and characterized a microfluidic device to reliably trap some *Arabidopsis thaliana* protoplasts and induced cell fusion by controlled addition of poly(ethylene glycol) (PEG, with a molecular weight of 6000). Experiments were conducted to determine the survival rate of isolated protoplasts in our microfluidic system. Afterward, PEG-induced fusion was studied. Our results indicate that the following fusion parameters had a significant impact on the fusion efficiency and duration: PEG concentration, osmolality of solution and flow velocity. A PEG concentration below 10% led to only partial fusion. The osmolality of the PEG fusion solution was found to strongly impact the fusion process; complete fusion of two source cells sufficiently took part in slightly hyper-osmotic solutions, whereas iso-osmotic solutions led to only partial fusion at a 20% PEG concentration. We observed accelerated fusion for higher fluid velocities. Until this study, it was common sense that fusion is one-directional, i.e., once two cells are fused into one cell, they stay fused. Here, we present for the first time the reversible fusion of protoplasts. Our microfluidic device paves the way to a deeper understanding of the kinetics and processes of cell fusion.

## 1. Introduction

Protoplasts are a versatile tool for plant cell biology [1]. For instance, they allow for transient transfection by osmotic shock with PEG-mediated plasmid import [2]. This method has been applied frequently for fluorescence microscopy: Multiple proteins have been localized this way, for instance V-ATPase subunits and malate [3,4]. Transient expression enabled the quantitative in vivo visualization of protein–protein interactions by bimolecular fluorescence complementation or Förster resonance energy transfer [4,5]. Transactivation of promoters by transcription factors has been addressed by using protoplasts, too [6]. Protoplasts are omnipotent cells that can be cultivated for callus formation or even plant growth, so protoplasting became a breeding tool in combination with cell–cell fusions and the formation of hybrid cells [7,8]. Cell fusions are regular processes in flowering plants, such as egg cell fertilization and synergid–endosperm fusion [9,10]. In the lab, fusion of protoplasts can be achieved by the addition of PEG or by applying an electric field [11]. The resulting hybrid cell carries organelles, transcriptomes, proteomes and genomes of two source cells, so analyzing the fate of all these turns cell fusion into a promising tool for the investigation of cellular organization and dynamics. Whereas the cellular organization subsequent fertilization and synergid–endosperm fusion is highly organized and regulated, the re-organization following protoplast fusion has to be either decisive or by chance. To investigate this process in detail, microfluidics is an appropriate and promising tool.

Standard devices like well plates are insufficient for the long-term observation of cells and single-cell observations. Microfluidic devices are versatile toolboxes for single-cell studies because of their small dimensions and the well-controlled environment [12]. For instance, the channel dimensions of the devices can be adapted according to the respective needs; in this work, the channel height has to be slightly larger than the protoplasts to provide long-term observations, without the cell diffusing out of the focus; this cannot be achieved with conventional lab dishes. Microfluidic devices provide access to single-cell investigations with time-lapse microscopy and thus yield inside views into the dynamics of processes [13]. Often, microfluidic devices are made monolithically from silicones that provide easy fabrication and tailoring of the devices` layout according to the respective studies [12]. So far, microfluidics has been used in numerous applications with plant cells [14]. For instance, microfluidic devices were used to conduct cultivation studies, providing deeper insight into cell mechanisms [12,15,16,17,18]. Microfluidics was also used to design small channels for the investigation of pollen and root growth in confined structures [19,20,21,22]. Sakai et al. used microfluidics to trap protoplasts over long time periods to observe cell wall reconstitution [23]. Ko et al. compared cell division rates of tobacco protoplasts in PDMS microfluidic devices and Petri dishes. They found that the microenvironment in the microfluidic devices did not harm the cells when cultured over several days and even provided better conditions for cell division than the Petri dish [24]. Wu et al. conducted tobacco protoplast fusion in a microfluidic device [25]. Their device consisted of a micro-column array that spanned the whole channel width, preventing the protoplasts from passing by. They added PEG of high concentration and observed fusion of cells that were randomly distributed [25]. To the authors’ knowledge, there has not been a microfluidic device that provides fusion of a controllable number of protoplasts, ideally two, so far. Here, we present for the first time a microfluidic device consisting of tailored features to trap two *A. thaliana* protoplasts in close contact, providing controlled PEG-induced fusion. This device was used to study the impact of various parameters impacting the fusion efficiency, duration and reversibility.

## 2. Materials and Methods

### 2.1. Buffers and Solutions

W5 buffer was used as a resuspension medium after the isolation of protoplasts. It contained 125 mM CaCl_2_, 5 mM KCl, 154 mM NaCl, 2 mM MES-KOH (pH 5.7) and 5 mM glucose. The cell wall was lysed enzymatically by cellulose R-10 and macerozyme R-10. The lysis solution contained 1.5% cellulase R-10, 0.4% macerozyme R-10 (both SERVA Electrophoresis, Heidelberg, Germany), 0.4 M mannitol, 20 mM KCl, 20 mM MES-KOH (pH 5.7), 10 mM CaCl_2_ and 0.1% BSA fraction V. Murashige & Skoog-media (MS-media) [26] was purchased from Duchefa (Haarlem, The Netherlands) without vitamins (M0221) and supplemented with Gamborg B5 vitamins [27].

The PEG fusion solution contained 0.147% CaCl_2_, PEG6000 of respective (*w*/*v*)% in W5 buffer and green fluorescent beads (0.5 µm FluoSpheres (505/515), Thermo Fisher Scientific, Dreieich, Germany) for staining the flow. After adjusting the pH value to 5.7 with KOH, the solution was autoclaved. Additionally, 500 µM F108 (BASF, Hanover, Germany) was added to all buffers used in the microfluidic devices serving as a dynamic surface-coating agent to suppress unspecific adsorption of the protoplasts to the channel surfaces and to increase the wettability of the hydrophobic PDMS. The surfaces of the PDMS became hydrophilic due to the surface coating [28], which also prevents the trapping of air bubbles.

### 2.2. Fabrication of Microfluidic Devices

The microfluidic devices were fabricated by PDMS (poly(dimethylsiloxane)) soft lithography as described in [29]. First, a silicon wafer was covered with SU-8(50) photoresist by spin coating at 3000 rpm for 30 s, leading to a structure height of 56 µm. Beforehand, the wafer was cleaned in caroic acid as described elsewhere. After spin coating, the photoresist was prebaked at 95 °C for 20 min and illuminated through a chromium mask (DeltaMask, Enschede, The Netherlands) for 29 s. The photoresist was hard-baked at 95 °C for 8 min before developing in an mr-dev developer (Microresist, Berlin, Germany) bath, submersion for 15 min and gentle rinsing every 2 min with acetone. After developing, the negative relief structures were visible on the master wafer, which was hard-baked before silanization with APTES (Sigma Aldrich, St. Louis, MO, USA).

PDMS elastomer (Sylgard 184, Dow Corning, Midland, MI, USA) was poured over the master wafer and thermally cured at 85 °C for 4 h. After curing, the PDMS was peeled off and reservoir holes were punched at the channel ends for fluidic access. The microfluidic channel was closed with a PDMS-covered glass slide after oxygen plasma treatment and filled with buffer.

### 2.3. Measurement Setup

A PMMA holder with 3 mm reservoir holes, to enlarge the reservoir volumes, was placed on the PDMS chip. Working buffer, PEG solution and protoplasts were pipetted into the respective reservoirs. The reservoirs were connected to a microfluidic flow control system (MFCS, Fluigent, Jena, Germany) via hoses. The microfluidic chip was then placed either on an inverted microscope, using an additional filter (U-25LBD, Olympus, Tokyo, Japan) to gain true-colored images, or a confocal laser scanning microscope (CLSM). The fluids and cells were driven through the microfluidic device by applying pressure in the range of 0.5 to 3.0 mbar.

### 2.4. Imaging

Cells in microfluidic devices were imaged with a bright-field microscope (Axiovert 100 TV, Zeiss, Oberkochen, Germany) with an illumination filter (U-25LBD, Olympus, Japan) and a CCD-camera (HCD-905, Day & Night digital color camera). Images were processed with Fiji (Version 1.53t). Additionally, microfluidic experiments were conducted on a CLSM (Zeiss LSM780 with 10-fold magnification). Images were obtained upon 488 nm excitation; the emission range was 500–600 nm for fluorescent beads and vacuolar staining and 650–700 nm for chlorophyll autofluorescence; the beam splitter was MBS488. In addition, bright-field images were obtained in the transmitted light mode. Images were processed with Zeiss Zen 3.7.

### 2.5. Protoplast Isolation, Purification and Fusion

Protoplasts were isolated as described before [30]. Briefly, approximately 2 g of four-week-old *A. thaliana* leaves were applied; this yields roughly 500,000 cells per mL or 6 million cells in total. The leaves were cut into leaf stripes, vacuum-infiltrated with lysis solution and incubated at room temperature for 2 h. Iso-osmotic solutions were achieved by adding 154 mM NaCl or 0.4 M mannitol to W5 solution, lysis solution or fusion solution, respectively. Cells were separated from debris by a nylon mesh and harvested by centrifugation at 100× *g* for three minutes. The viability tests were conducted either by detecting the chlorophyll absorption at 650–700 nm as a marker for viability or by vital staining with neutral red.

The fusion was induced by flushing the trapped cells with a PEG fusion solution of respective concentrations.

### 2.6. Statistical Analysis

For statistical analyses, means and standard deviations were calculated, and Student’s *t*-test was performed as indicated in the figure legends.

## 3. Results

### 3.1. Microfluidic Device Fabrication and Characterization

When the microfluidic devices were designed, we had to consider the size of the protoplasts before and after fusion; the latter was estimated because this directly set the bottom limit of the channel height and the dimensions of the trapping features. Protoplasts of *A. thaliana* are about 30 µm in diameter. Also taking into account that some additional space had to be around the protoplast to enable sufficient flushing of both medium and protoplasts, we set the channel height to a minimum of 55 µm. The minimum width of the trapping features was set to 90 µm to enable easy trapping of protoplasts. We observed that the trapping of protoplasts in shallower features took longer because of directing the cells into the shallow trap entrance.

**Figure 1:**
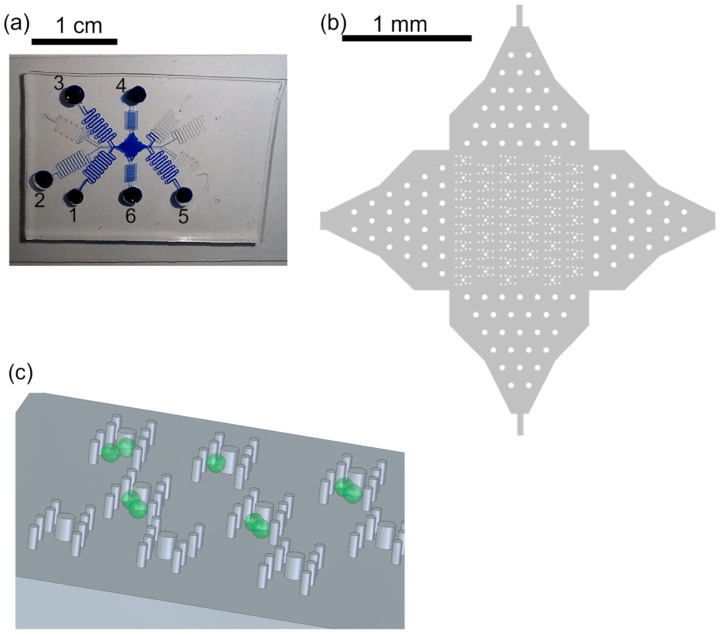
Trapping features. (**a**) Photograph of device filled with blue ink for better visualization. The protoplasts were in reservoir 1, W5 buffer was in reservoirs 2, 4–6. PEG fusion buffer was in reservoir 3. The channel lengths were about 20 cm to gain flow control. (**b**) Overview of array of trapping regions with posts. The posts in the channel entrances served for pre-distribution of cells and better flow control. (**c**) Sketch of posts (not to scale), aligned in H-shape to form the trapping features, and cells (green). The posts reach from channel bottom to channel ceiling, 56 µm.

The first trapping features of the protoplasts were made of posts, reaching from channel bottom to ceiling, arranged in a double U-shape (Figure 1). We tested two diameters, 20 µm and 40 µm, of the posts and evaluated them with respect to the ease of fabrication and the fluid behavior in the final device. The microfluidic device was fabricated by PDMS soft lithography; see Section 2.2, where the channel structure was cast from a master wafer. During the fabrication of the master wafer, the recesses in the photoresist from which the posts were made were the largest challenge; this was because the removal of the uncured photoresist from the recess was difficult since the developer hardly penetrated that region and removed the resist and residuals remained in that area. This was intensified for photoresist layers of larger thicknesses.

We improved the photoresist development process by gently shaking the beaker with the wafer in the developer every minute starting after 8 min of developing. Therefore, the photoresist was removed from the post recesses and intact post structures could be molded from the master wafer with PDMS (Figure 1).

After the fabrication of the microfluidic device, we evaluated the fluid stream with a special focus on the region of the trapping features. For that purpose, we ran two fluid streams, one with pure water and one with the fluorescent dye fluorescein isothiocyanate (FITC), and monitored the streams using fluorescent microscopy. Although the typical laminar flow could be observed in the channel, the posts of smaller diameter led to less disturbance of the fluid stream than the 40 µm posts. Therefore, we used posts with a 20 µm diameter for all subsequent protoplast experiments.

Appropriate flow control via pressure-driven flow was significantly improved by using a channel length of 20 cm. This was necessary because of the large channel cross-section, the channel width of 300 µm and the height of 56 µm, and thus low flow resistance.

**Figure 2:**
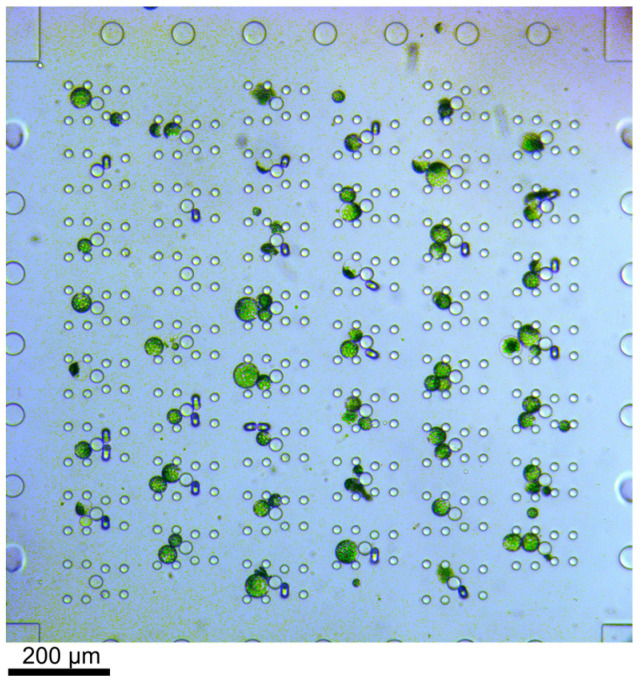
Bright-field microscopy image of trapped protoplasts. The cells were flushed through the device from left to right. Excess cells, i.e., if more than two cells were trapped in one trap, were removed by applying flow from the top to the bottom through the array or vice versa. The channel height was 56 µm and thus larger than the cells. Therefore, fluids were never completely blocked by cells in the trap.

Protoplasts were directed through the microfluidic device by applying sufficient pressure to the respective reservoirs; see Figure 1. The flow of the cells could be easily controlled due to the laminar flow; thus, the stream of cells in the trapping array was adapted by applying additional flow perpendicular to the main flow direction, i.e., pressure was applied either to reservoir 4 or 6 to ensure most of the traps were filled with two protoplasts. Thus, the filling efficiency was not solely dependent on the cell distribution in the flow but could be controlled. Once protoplasts entered the trapping feature, they were reliably withheld by the posts (see Figure 2), i.e., trapped, until the fluid stream was inverted. Excess cells, i.e., if more than two cells were trapped, were removed by a flow perpendicular to the trapping features.

### 3.2. Determination of Cell Vitality

Protoplast fusion is followed by a (re-)organization of the hybrid cell, which is expected to take at least a few hours. Therefore, the vitality of cells was monitored in the microfluidic device over time to test the vitality in the microfluidic devices. Cells were stained with neutral red as a vital stain and the time point of the loss of the red staining was monitored (Supplementary Figure S2). Then, the shape of the cells was analyzed. Neutral red destaining occurred after 212 ± 68 min (mean ± SE, *n* = 7) and the cells collapsed after 663 ± 141 min (mean ± SE, *n* = 7). Cooling of the device resulted in no significant alteration of the cellular vitality (*t*-test, *p* = 0.83).

Additionally, the vitality of the cells in the microfluidic devices was analyzed by visual inspection, i.e., without staining so as not to interfere with the fusion process, during cell fusion. The analysis revealed that the cells kept their shape and size with small deviations, and thus, the fused protoplasts were assumed to be alive during the whole process, i.e., trapping, fusion and observation afterward.

### 3.3. PEG Concentrations for Fusions—Batch Experiments

**Figure 3:**
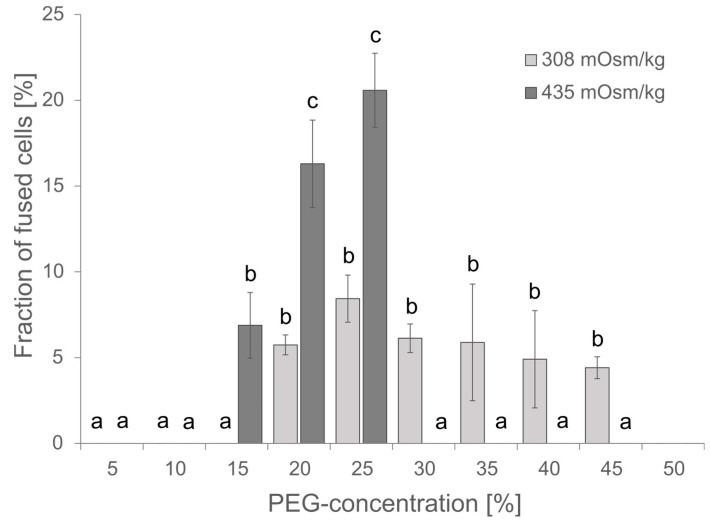
Effect of PEG concentration and osmolality on the fusion efficiency. For each PEG concentration, 80–300 protoplasts were analyzed for apparent cell fusions either in NaCl media of 308 mOsm kg^−1^ or in 0.4 M Mannitol 2 mM MES pH 5.7 of 435 mOsm kg^−1^. Mean ± SE is given, *n* = 3 independent counts. Significance was proven by ANOVA followed by Scheffe post hoc test. Identical letters mark data that are not significantly different while different letters stand for significantly different datasets (*p* < 0.05).

The PEG concentration is critical for the fusion efficiency, but PEG might also be cytotoxic at higher concentrations of more than 25% as observed for the transfection of protoplasts from *Elaeis guineensis* [31]. Although the PEG concentration has been estimated for protoplast fusions before by Xiao and co-workers, and within their studies, 20% PEG gave the best results [32], the conditions might be different within a microfluidic device. Vitality of cells and fusion efficiency have been investigated for PEG concentrations in the range of 0 to 45%. Fusion efficiency has been defined as the ratio of fused cells to single cells; fused cells showed at least a partial merging of two spherical cells. Cells that showed simply a punctual membrane contact were considered not fused. The highest fusion efficiency was observed at 20–25% PEG, and the survival of cells was unaffected at PEG concentrations ≤ 20% as indicated by insignificant changes in chlorophyll absorption (Supplementary Figure S1). Therefore, subsequent experiments were started with 20% PEG. Usually, 0.4 M mannitol or 154 mM NaCl are used as osmotically active components in Arabidopsis media, though both differ in their osmolality of 435 and 308 mOsm kg^−1^, respectively. Comparing the effect of osmolality on cell fusions revealed increased toxicity for higher osmolality and a resulting lethality at PEG concentrations of 30% and higher but also an improved fusion rate and thus, more efficient fusion at 20–25% PEG and 435 mOsm kg^−1^ (Figure 3).

### 3.4. Characterization of PEG-Induced Fusion in Microfluidic Devices

After fabrication and characterization of the microfluidic device for trapping protoplasts, we tested different PEG concentrations for the induction of fusion of protoplasts. We flushed the microfluidic device with protoplasts until two to three protoplasts were trapped. The applied protoplast suspension had a concentration of roughly 500,000 cells per mL or 6 million cells in total.

Protoplast fusion was induced by introducing a PEG buffer into the trapping array after sufficient loading of the trapping features with protoplasts. We studied PEG-induced fusion at varying concentrations to determine the minimum concentration at which reliably induced fusion was possible. This was conducted because PEG is known to be cytotoxic [33]. We tested concentrations from 30% down to 7.5%.

**Figure 4:**
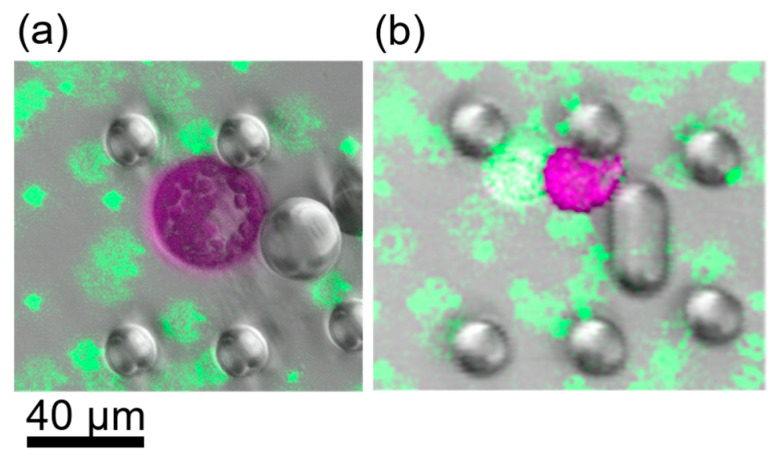
CLSM images of cells after induction of fusion. Images display false colours, Chloroplast fluorescence is depicted in purple, green fluorescence in green. (**a**) Cells after fusion with 13.75% PEG. Green fluorescent beads were used as dye to stain the PEG fusion solution. The shape is perfectly spherical. (**b**) Fusion induction with PEG concentrations less than 10% led to partial fusion, i.e., the cells stuck together but did not exhibit a spherical shape.

Reliable fusions were observed for all PEG concentrations of 10% and higher. The fused cells exhibited a perfectly spherical shape (see Figure 4a). At PEG concentrations of 8.75% and 7.5%, only partial fusions were observed. For instance, two cells could be distinguished by eye as the partially fused cells did not reach a state of spherical shape (see Figure 4b).

The fusion experiments were conducted at various flow velocities (9–32 µm/s) to test which led to the best performance. The fusion duration increased for slower flows, whereas at higher flows, the cells were more often squeezed through the small gaps between the post, and thus escaped from the trap after PEG reached the cells. The best velocity was found to be about 20 µm/s, which was used for subsequent experiments.

To gain further insight into the fusion process and its relevant parameters, we determined the sizes of the source cells and fused cells and calculated the size differences (see Supplementary Table S1). Those measurements revealed that the fused cells were smaller than expected, based on the volume of the source cells. This was in agreement with observations during the fusion; cells that came in contact with the PEG solution immediately shrunk. Interestingly, we found that fusion always started about 35 s after the PEG fusion solution reached the source cells. That is, the two source cells started to move into each other (see Supplementary Video S1).

The osmolality of the PEG solution was regulated by additional mannitol. However, we also tested a PEG solution with 154 mM NaCl to regulate the osmolality as that was used for the resuspension solutions as well. Fusion experiments with those solutions and a PEG concentration of 20% only led to partially fused cells, similar to those in Figure 4b. Furthermore, the cells did not change their volume when the PEG solution reached them but remained constant.

**Figure 5:**
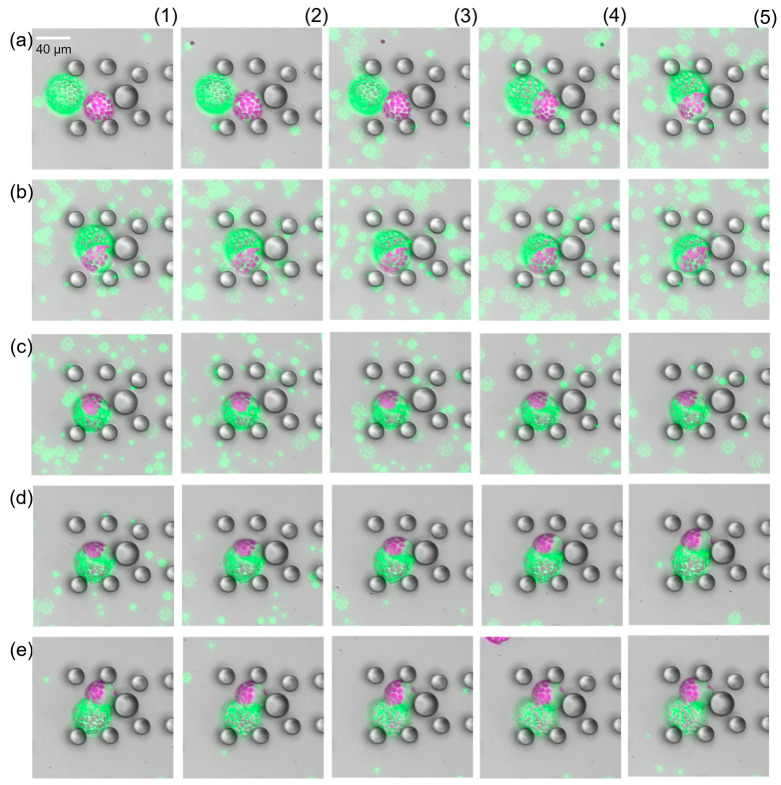
Time-lapse study of PEG-induced protoplast fusion (**a1**–**b5**) and separation (**c1**–**e5**). Images display false colours, Chloroplast fluorescence is depicted in purple, green fluorescence in green. There were 15 s time intervals between consecutive images. Impact of PEG on the source cells was observed about 35 s after first contact. After successful fusion image (**b5**), the PEG fusion solution was washed away (**c1**–**e5**). The two source cells fully separated after removal of the PEG fusion solution.

Having close looks at the fully fused cells, we found structures that looked like the cells remained separated by a membrane-like structure see Supplementary Figure S3. First, we assumed that the new intracellular structure was a relic of previous cell membranes, which were highly permeable or fragmented and thus provided the perfect spherical shape. But this was proven wrong after flushing the fused cells with resuspension buffer. As soon as the PEG fusion solution started being replaced, the fused cells started to separate again into the source cells (Figure 5). In all cases, we first observed a swelling of the fused cell followed by an excrescence of one source cell. This process was slower than the fusion but happened in all cases. Fusion and separation could be repeated as long as both source cells were kept intact (see Supplementary Video S1). This was absolutely unexpected and has not been documented in the literature so far. Analysis of the protoplast radii before fusion and after separation revealed that the size was the same, within the expected measurement error margin.

Although we focused the fusion on the analysis of two fused cells, even three cells fused. In any case, for both two-cell-fused and three-cell-fused protoplasts, a rotation due to the fluid flow was observed, though it was far slower for the three-cell-fused protoplasts. Determination of the diameter of the fused cells revealed that it was almost as large as the channel height for the three-cell-fused ones. Therefore, rotation was hindered.

## 4. Discussion

The microfluidic devices used in this work were fabricated monolithically with PDMS soft lithography. Fabrication of the negative relief structure, the trapping features, on the master wafer was challenging due to the small diameter of the recesses, which had a diameter of 20 µm and a depth of 56 µm. Adaptation of the development process with gentle shaking of the beaker with the wafer led to good penetration of the developer into the recesses and thus removed the photoresist. Though this high aspect ratio of 2.8 was achieved due to optimized fabrication, this states a current limitation towards fabricating higher microfluidic devices. Therefore, other photoresists might be tested for the master wafer in future studies.

The vitality of the cells was maintained in the microfluidic devices for at least three hours as observed by vital staining; analyzing the shape pointed to even ten hours without much adjustment of the conditions and media supply. These data demonstrate that the setup is suitable for long-term observations of subsequent fusion and allows for investigations of cellular re-organization. The addition of up to 25% PEG did not significantly reduce the vitality of cells in the microfluidic device. This is in very good accordance with previous literature, where microfluidics were used to study pollen and root growth [19,20,21,22].

Successful protoplast fusion was conducted in the microfluidic devices after trapping two protoplasts. This was the first time the controlled fusion of two protoplasts was demonstrated. Previously, Wu et al. conducted PEG-induced fusion experiments of randomly distributed tobacco protoplast fusion in a microfluidic device [25]. Their device consisted of a micro-column array that spanned the whole channel width, preventing the protoplasts from passing by. We observed that the fusion of the protoplasts depended on (1) the PEG concentration of the fusion buffer, (2) the flow velocity and (3) the osmolality.

(1) PEG concentration had an impact on both the duration of the fusion process and whether the fusion process was complete or partial. PEG in the vicinity of a membrane removes the hydration layer that impedes the close apposition of converging phospholipid bilayers [34]. We assume that this process was independent of the PEG concentration since the first cell approaching and merging was observed after about 35 s for all PEG concentrations tested. PEG is assumed to induce fusion by causing small damages in the cell membrane [35]. Those regions, when in contact with a second cell, might serve as a starting point for fusion. PEG damage of membranes is assumed to be concentration-dependent as diffusion and integration of PEG into the cell membrane is driven by the PEG concentration gradient, theoretically described by Fick’s law [36]. This is in very good agreement with our experimental findings that the total duration of fusion was longer for lower PEG concentrations. Additionally, a minimum concentration of 10% PEG was found in the microfluidic experiments. Only partial fusion was observed if the concentration was below 10% PEG. Hence, we could demonstrate successful fusion with reduced PEG concentrations compared to previous experiments and batch experiments off-chip.

(2) We assume the fusion velocity depends on the flow velocity due to better contact of the two source protoplasts being pressed against each other and a shearing of the two membranes in close contact improving the membrane opening for fusion.

(3) Analysis of the radii of source cells and fused cells revealed that the fused cells’ volume was smaller than expected. This was in accordance with the osmolality of the PEG fusion solution, in which we set the osmolality with mannitol, and the resuspension buffer contained NaCl to set the osmolality. The latter was of lower osmolality (308 and 435 mOsm/kg for the resuspension buffer and fusion solution, respectively) and thus, the turgor decreased when the cells were flushed with PEG fusion buffer. We assume that osmolality impacts the fusion efficiency, since fusion experiments with NaCl in the PEG fusion solution, i.e., being iso-osmotic, revealed that the radii remained constant and that no complete fusion took part. Higher osmolality of the fusion buffer led to reduced turgor in the source cells, and thus enabled better nestling of the cells against each other, providing improved cell contact. Additionally, reduced turgor and cell radii led to increased membrane curvature. Malinin et al. reported that vesicle radius strongly impacts the mixing of the vesicles’ lipids [37]. Though protoplasts are not vesicles, their membranes can be compared with each other and so can their fusions or lipid-mixing behaviors. For this reason, we propose to use slightly hyperosmotic fusion solutions to improve fusion efficiency. Additionally, the separation of fused cells after the removal of the hyperosmotic buffer also indicates that the fusion strongly depends on the osmotic strength, and thus the turgor. The separation of the fused cells occurred in all experiments after the removal of the mannitol PEG buffer. The separation of the fused cells also indicated that the fusion process was not completed within minutes but likely needed more time to fully merge the two membranes into one. We assume that separation takes place as long as two vacuoles remain in the cell, i.e., do not fuse into one. This needs to be proven in future studies.

A significant difference in fusion efficiency was found in our study comparing the fusion efficiency in microtiter wells, i.e., batch, and microfluidics. The batch experiments reached a maximum fusion efficiency of about 20%, whereas the experiments in our microfluidic device had a fusion efficiency of more than 95%.

In rare events, we observed the fusion of three source cells, though in most cases, we flushed away additional cells excessing two trapped cells. The fusion of two protoplasts was faster than fusion of three protoplasts. We assume that this was due to the fact that the PEG more easily reaches larger parts of the cell membrane for two trapped protoplasts. Additionally, the channel height of 56 µm limited the cell rotation of fused cells if they were too large in diameter, also perturbing successful fusion.

## 5. Conclusions and Outlook

In this work, we designed and fabricated a monolithic microfluidic device consisting of trapping features to reliably trap two *A. thaliana* protoplasts at a time. The trapping features were designed such that the protoplasts were in close contact but could, at the same time, be flushed with media. This enabled us to demonstrate (for the first time) reliable PEG-induced fusion of two *A. thaliana* protoplasts in a microfluidic device. The experiments revealed that the fusion and its duration strongly depend on the PEG concentration, the osmolality of the fusion solution and the applied flow velocity, i.e., shear forces. Additionally, our microfluidic device provided new insight into the reversibility of the fusion process. We observed the separation of a fused cell in source cells after washing away the PEG fusion solution; this has not been reported in the literature before.

In the future, we will conduct further experiments to gain a deeper understanding of PEG-induced fusion. For instance, different fluorescent labeling of the vacuole and cell nucleus can be used to study if those also fuse into one vacuole and one nucleus. Additionally, future studies need to focus on the minimum concentration of PEG and exposure times that provide complete fusion and formation of two permanently fused cells. The experiments presented in this work were conducted with homotype cells, though the device also yields the opportunity to fuse heterotype cells. For that purpose, first cell type 1 will be trapped, followed by a transfer of the cells into the opposite trap and filling with cell type 2 (see Supplementary Figure S4).

## Data Availability

All data generated or analyzed during this study are included in this published article and its supplementary information files. The datasets used and/or analyzed during the current study are available from the corresponding author on reasonable request.

## Supplementary Materials

The following supporting information can be downloaded at https://www.mdpi.com/article/10.3390/plants13020295/s1, Figure S1: Insignificant changes in chlorophyll absorption indicate survival of cells was unaffected at PEG concentration ≤ 20%; Figure S2: The vitality of cells was monitored in the microfluidic device over time to test the vitality in the microfluidic devices. Cells were stained with neutral red as vital stain and the time point of the loss of the red staining was monitored. (a) Cell at time 0 min. (b) This cell was destained after 336 min; Figure S3: Zoom on fused cell. A close look revealed that the two source cells are separated by a membrane-like structure (indicated by arrow); Figure S4: Sketch of heterotype cell trapping in the asymmetrically sized traps; the right trapping area is smaller to host just one cell, whereas the left trapping area is large enough to host two cells. (a) The trapping of heterogeneous cells could be performed by consecutively filling the traps with cell type 1, green, from the right (arrow indicating the direction of flow), (b) followed by filling the traps with cell type 2, yellow, from the left. The cells trapped in the first step are transferred into the opposite trap during the second step; Table S1: Change in radius of fused cells compared to the source cells depending on the PEG concentration. The expected radius is calculated based on the radii before fusion and assuming that the total volume remains constant; Video S1: Induced fusion (with 15% PEG) and separation. First, two source cells are trapped and fused. After removal of PEG, the cells separated. After trapping a third cell, again fusion was induced by PEG flushing and all three cells fused into one cell. Removal of PEG led to separation into three source cells.
