# Supplementary material for: Microfluidic Single-Cell Study on Arabidopsis thaliana Protoplast Fusion—New Insights on Timescales and Reversibilities
Source: Plants (Basel). 2024 Jan 18;13(2):295. doi: 10.3390/plants13020295 (PMC10820889; doi:10.3390/plants13020295)
Supplement: Supplementary file 1 [file plants-13-00295-s001.zip › plants-2797357-supplementary.pdf]

Supplementary information

Microfluidic Single Cell Study on *Arabidopsis thaliana* Protoplast Fusion – New Insights on Timescales and Reversibilities

Thorsten Seidel [1,*], Philipp Johannes Artmann [2], Ioannis Gkekas [2], Franziska Illies [1,2], Anna-Lena Baack[1], and Martina Viefhues [2,*]

[1] Dynamic Cell Imaging, Faculty of Biology, Bielefeld University, 33615 Bielefeld, Germany
[2] Experimental Biophysics and Applied Nanosciences, Faculty of Physics, Bielefeld University, 33615 Bielefeld, Germany
* Correspondence: thorsten.seidel@uni-bielefeld.de (T.S.); viefhues@physik.uni-bielefeld.de (M.V.)

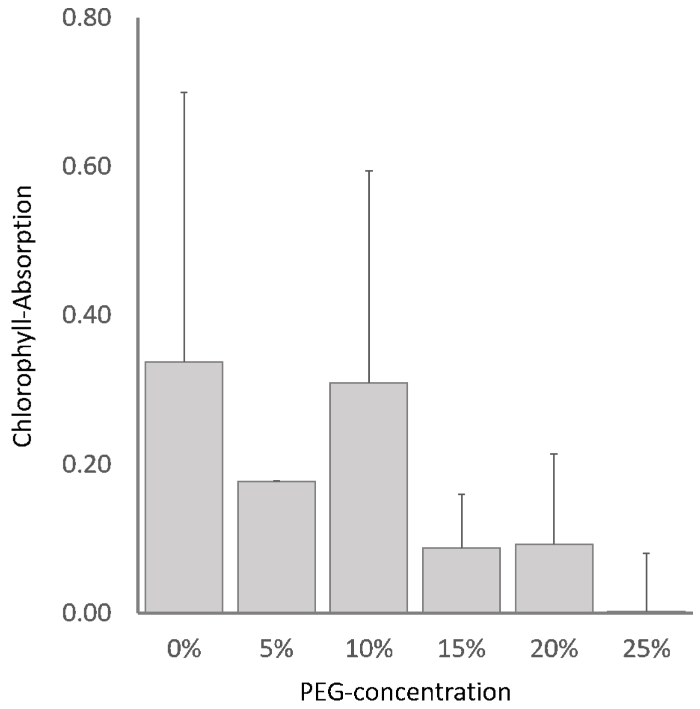

Supplementary Figure S1: insignificant changes in chlorophyll absorption indicate survival of cells was unaffected at PEG-concentration ≤ 20 %.

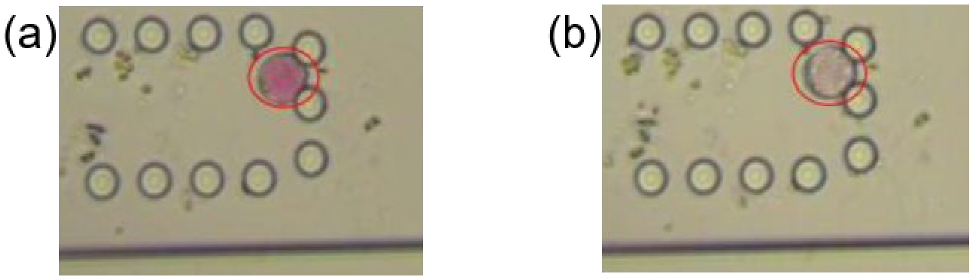

Supplementary Figure S2: the vitality of cells was monitored in the microfluidic device over time to test the vitality in the microfluidic devices. Cells were stained with neutral red as vital stain and the time point of the loss of the red staining was monitored. (**a**) Cell at time 0 min. (**b**) This cell was destained after 336 min.

Table S1: Change of radius of fused cell compared to the source cells depending on the PEG concentration. The expected radius is calculated based on the radii before fusion and assuming that the total volume remains constant.

PEG Concentration [%]	Size Variation from Expectation [%]	Variance Size Expectation
10	20.74	9.82
12.5	25.82	3.81
13.75	32.77	4.70
15	37.79	13.57
17.5	37.56	5.99
18.75	39.44	13.25
20	35.52	16.52
25	48.42	17.47
30	38.96	36.34

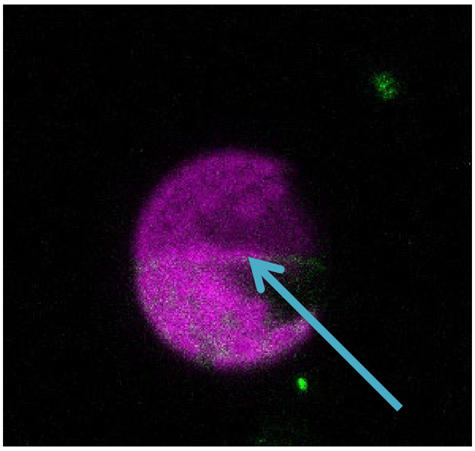

Supplementary Figure S3: Zoom on fused cell. A close look revealed that the two source cells are separated by a membrane like structure (indicated by arrow).

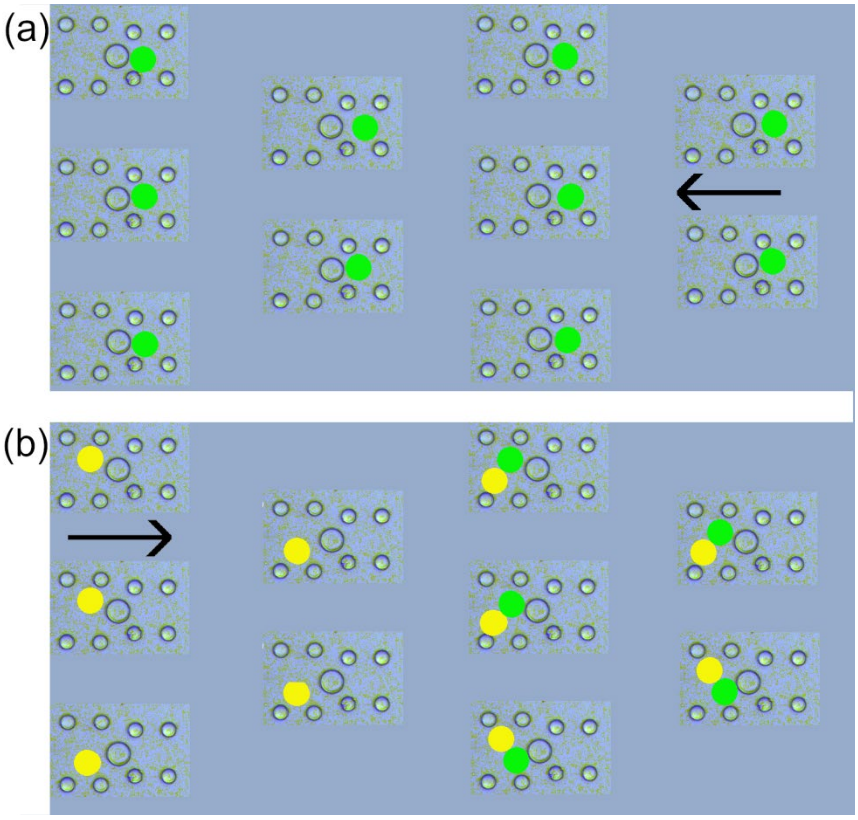

Supplementary Figure S4: Sketch of heterotype cell trapping in the asymmetrically sized traps, the right trapping area is smaller to host just one cell, whereas the left tapping area is large enough to host two cells. (**a**) The trapping of heterogeneous cells could be done by consecutively filling the traps with cell type 1, green, from the right (arrow indicating the direction of flow); (**b**) followed by filling the traps with cell type 2, yellow, from the left. The cells trapped in the first step are transferred into the opposite trap during the second step.
